# Lung function and atherosclerosis: a cross-sectional study of multimorbidity in rural Uganda

**DOI:** 10.1186/s12890-021-01792-0

**Published:** 2022-01-05

**Authors:** Rebecca F. Gilbert, Cody Cichowitz, Prossy Bibangambah, June-Ho Kim, Linda C. Hemphill, Isabelle T. Yang, Ruth N. Sentongo, Bernard Kakuhikire, David C. Christiani, Alexander C. Tsai, Samson Okello, Mark J. Siedner, Crystal M. North

**Affiliations:** 1 Massachusetts General Hospital, 55 Fruit Street, BUL-148, Boston, MA 02114 USA; 2 Mbarara University of Science and Technology, Mbarara, Uganda; 3 Brigham and Women’s Hospital, Boston, MA USA; 4 Harvard Medical School, Boston, MA USA; 5 Geisel School of Medicine at Dartmouth, Hanover, NH USA; 6 Harvard TH Chan School of Public Health, Boston, MA USA

**Keywords:** COPD, Cardiovascular disease, HIV infection, FEV_1_, cIMT, Uganda

## Abstract

**Background:**

Chronic obstructive pulmonary disease (COPD) is a leading cause of global mortality. In high-income settings, the presence of cardiovascular disease among people with COPD increases mortality and complicates longitudinal disease management. An estimated 26 million people are living with COPD in sub-Saharan Africa, where risk factors for co-occurring pulmonary and cardiovascular disease may differ from high-income settings but remain uncharacterized. As non-communicable diseases have become the leading cause of death in sub-Saharan Africa, defining multimorbidity in this setting is critical to inform the required scale-up of existing healthcare infrastructure.

**Methods:**

We measured lung function and carotid intima media thickness (cIMT) among participants in the UGANDAC Study. Study participants were over 40 years old and equally divided into people living with HIV (PLWH) and an age- and sex-similar, HIV-uninfected control population. We fit multivariable linear regression models to characterize the relationship between lung function (forced expiratory volume in one second, FEV_1_) and pre-clinical atherosclerosis (cIMT), and evaluated for effect modification by age, sex, smoking history, HIV, and socioeconomic status.

**Results:**

Of 265 participants, median age was 52 years, 125 (47%) were women, and 140 (53%) were PLWH. Most participants who met criteria for COPD were PLWH (13/17, 76%). Median cIMT was 0.67 mm (IQR: 0.60 to 0.74), which did not differ by HIV serostatus. In models adjusted for age, sex, socioeconomic status, smoking, and HIV, lower FEV_1_ was associated with increased cIMT (β = 0.006 per 200 mL FEV_1_ decrease; 95% CI 0.002 to 0.011, *p* = 0.01). There was no evidence that age, sex, HIV serostatus, smoking, or socioeconomic status modified the relationship between FEV_1_ and cIMT.

**Conclusions:**

Impaired lung function was associated with increased cIMT, a measure of pre-clinical atherosclerosis, among adults with and without HIV in rural Uganda. Future work should explore how co-occurring lung and cardiovascular disease might share risk factors and contribute to health outcomes in sub-Saharan Africa.

**Supplementary Information:**

The online version contains supplementary material available at 10.1186/s12890-021-01792-0.

## Background

Chronic obstructive pulmonary disease (COPD) and cardiovascular disease—leading causes of global morbidity and mortality—are responsible for nearly 22 million deaths yearly [1]. Data from high income settings demonstrate that people with COPD have a more than twofold increased risk of concomitant cardiovascular disease [2, 3], which contributes substantially to disease-related health and economic impacts, complicates longitudinal disease management, and increases overall mortality [4, 5]. Similarly, reduced lung function is one of the strongest predictors of mortality among people with cardiovascular disease [6]. In sub-Saharan Africa, an estimated 26 million people are living with COPD [7] and one million people die from cardiovascular disease each year [8], yet little is known about the prevalence, risk factors, or health outcomes of concomitant cardiovascular disease among those with lung disease in the region.

COPD and cardiovascular disease share several risk factors, all of which center upon states of persistent systemic inflammation [9–11]. However, differences in the epidemiology of these risk factors in sub-Saharan Africa suggest that our understanding of co-occurring COPD and cardiovascular disease derived from data collected in high income settings may not be generalizable. Smoking, for example, is the leading cause of both lung and cardiovascular disease globally [12]. However, smoking is less prevalent in many regions of sub-Saharan Africa as compared to high-income settings [13, 14], and how this influences risk of concomitant lung and cardiovascular disease remains to be seen. On the other hand, air pollution—the greatest environmental threat to health—is causally associated with both lung and cardiovascular disease [15], is more prevalent across sub-Saharan Africa as compared to high income settings [16], and may further heighten the risk of cardiovascular disease mortality among people with COPD [17]. Lastly, most of the 38 million people with HIV globally live in sub-Saharan Africa [18]. Data from high income settings suggest that chronic HIV infection independently increases the risk of both COPD and cardiovascular disease [19, 20], and while relationships between HIV and COPD seem to be similar in African populations [21–24], emerging data suggests that PLWH in sub-Saharan Africa may have a different cardiovascular disease risk profile than their counterparts in high income settings [25, 26]. Thus, HIV may influence relationships between lung and cardiovascular disease risk differently in HIV endemic regions, but these patterns and their implications for disease prevalence and health outcomes have not been well-characterized.

As life expectancy on the African continent continues to improve [27–29], non-communicable diseases have become a leading source of morbidity and mortality [30–32]. As a result, defining multi-morbidity in sub-Saharan Africa is crucial step in establishing the next generation of public health priorities and scaling-up the existing healthcare infrastructure to meet this as-of-yet unmeasured need [33–35]. To address this gap in knowledge, we characterized the relationship between lung function and cardiovascular disease risk, and evaluated for potential effect modification by age, gender, smoking history, HIV serostatus and socioeconomic status, in a mixed cohort of adults with and without HIV in southwestern Uganda.

## Methods

### Study design, population, and setting

The data for this cross-sectional study were collected as part of the Uganda Non-Communicable Diseases and Aging Cohort (UGANDAC) Study (NCT02445079), an observational cohort study of older adults with and without HIV that has been described in detail previously [25, 36, 37]. Briefly, PLWH were eligible for enrollment if they were at least 40 years of age, had been taking antiretroviral therapy for at least three years, and were receiving care at the HIV clinic at the Mbarara Regional Referral Hospital. A sex and age-similar (matched by quartiles of age) population-based cohort of HIV uninfected participants was recruited from a complete population census from a community within the clinical catchment area [38] and were confirmed to be HIV negative before each study visit.

### Data collection, exposures and outcome

Participants completed annual study visits from August 2015 through May 2018, during which study staff collected demographic and health data using structured survey instruments, measured post-bronchodilator lung function with handheld spirometry, and measured carotid intima media thickness (cIMT) with ultrasonography. We characterized socioeconomic status with the method developed by Filmer and Pritchett [39], which uses principal components analysis to define quartiles of wealth within the study population based on a series of 21 questions about household assets and living conditions [40]. We defined smoking status using the WHO STEPS questionnaire [41], and defined biomass exposure as the type of cooking fuel used in the participant’s home. For PLWH, we obtained data on most recent CD4 count, viral load, and antiretroviral therapy regimen through clinical record abstraction from the most recent HIV clinic visit within the last 12 months. We defined viral load suppression as a viral load below the limit of assay detection (dried blood spot: < 550 copies/µL; plasma: < 40 copies/µL; Roche Cobas® assay, Pleasanton, CA).

Our primary outcome of interest was pre-clinical atherosclerosis, which we defined using cIMT, an ultrasound-based measure of the thickness of the carotid artery that has been associated with atherosclerosis and cardiovascular disease risk [25, 42]. cIMT was measured by two operators (PB and JHK) who completed training at the University of Wisconsin Carotid Intima Media Thickness Course, using a Sonosite M-Turbo machine (Sonosite, Bothell, WA). In accordance with American Society of Echocardiography guidelines, sonographic images were obtained in the anterior, lateral, and posterior positions of both the right and left common carotid artery at one centimeter proximal to the carotid bulb using a semi-automated edge-detection software platform (SonoCalc, Version 5.0, Sonosite, Bothell, WA). Each participant had a total of six cIMT measures per visit, which were measured by a single reader (IY) and scored for quality by the study vascular cardiologist (LCH). We calculated cIMT for each participant as the average value of all measurements that met prespecified quality standards [43].

Our primary explanatory variable of interest was lung function, which we defined as post-bronchodilator forced expiratory volume in one second (FEV_1_), which represents the amount of air expelled into the environment in the first second of exhalation using maximum effort. This was measured using spirometry and the methodology described previously [21, 36]. Participants completed handheld spirometry in the seated position with the instruction of trained study staff using EasyOne® Plus handheld spirometers (ndd Medical Technologies Inc., Andover, MA) in accordance with American Thoracic Society (ATS) guidelines [44]. Measured lung function parameters included FEV_1_ and forced vital capacity (FVC). Participants with an FEV_1_ to FVC ratio less than 0.7 were asked to inhale four puffs of albuterol (Ventolin, GlaxoSmithKline, Philadelphia, PA) and rest for 10 minutes*,* after which spirometry was repeated. Spirometry was evaluated for acceptability and reproducibility using ATS guidelines [45] and interpreted using NHANES III prediction equations [46]. We defined COPD as a post-bronchodilator FEV_1_ to FVC ratio less than 0.7 and graded the severity of obstruction using the Global Initiative for Chronic Obstructive Lung Disease (GOLD) criteria [47].

### Statistical analysis

We included data from the first study visit at which a participant completed both spirometry and carotid ultrasound measurements. We summarized cohort characteristics using Mann–Whitney U (rank sum), chi squared, or fisher’s exact tests as appropriate. We evaluated for selection bias by comparing the characteristics of study participants who had concomitant spirometry and cIMT measurements versus those who did not have concomitant measurements, and compared the characteristics of study participants with ATS-acceptable spirometry results versus those whose spirometry results did not meet ATS acceptability and reproducibility guidelines. We then fit linear regression models adjusted for age, sex, smoking history, HIV serostatus, and socioeconomic status to characterize the relationship between FEV_1_ and mean cIMT. We pre-specified the inclusion of these covariates in the models based on their known relationships with both obstructive lung disease and atherosclerosis. We did not include biomass exposure in the models because biomass exposure was ubiquitous across the cohort. We evaluated whether age, sex, smoking history, HIV, or socioeconomic status were effect modifiers of the relationship between FEV_1_ and cIMT by including a FEV_1_-by-covariate product term in each respective model. We also compared the FEV_1_ coefficient in regression models stratified by each covariate of interest. To evaluate the robustness of our findings, we conducted a sensitivity analysis in which we defined lung function using FEV_1_/FVC rather than FEV_1_. A final sensitivity analysis was preformed that included physical activity in the model, given its known association with lung and cardiovascular disease. Analyses were completed using Stata (Version 15, StataCorp, College Station, TX).

### Ethics statement

All participants gave written informed consent. Study protocols were approved by the ethics review committees at Mbarara University of Science and Technology and Partners Healthcare, the Uganda National Council for Science and Technology, and the Research Secretariat in the Office of the President (Uganda).

## Results

**Fig. 1:**
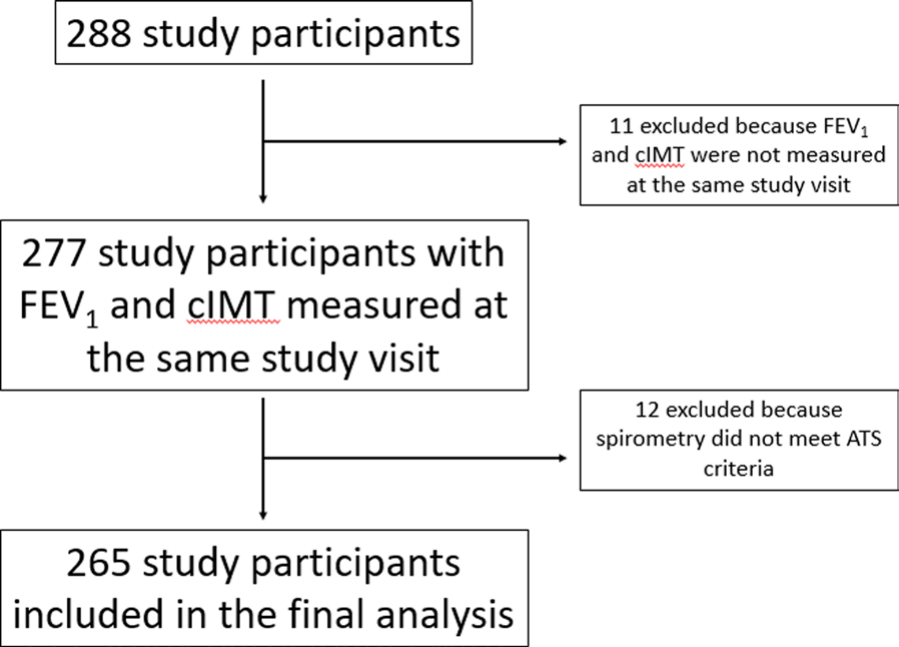
Flow diagram of study participant enrollment

A total of 288 participants completed at least one study visit between August 11, 2015 and May 7, 2018. Of these, 277 (96%) had both spirometry and carotid ultrasonography completed at the same study visit. An additional 12 (4%) of the 277 participants were excluded because their spirometry did not meet ATS acceptability or reproducibility standards, for a total analytic cohort of 265 participants (Fig. 1). The 11 participants who did not have both spirometry and carotid ultrasound measurements during the study period self-reported a history of hypertension more often than those with both measurements (45% versus 19%, respectively; *p* = 0.05). Otherwise, there were no substantive or statistical differences between participants who had concomitant spirometry and carotid ultrasound as compared to those who did not (Additional file 1: Table S1), or between those whose spirometry met ATS guidelines as compared to those with ATS-unacceptable spirometry (Additional file 1: Table S2).

Among the 265 participants in the analytic dataset, 125 (47%) were women, 140 (53%) were PLWH, and primary school was generally the highest level of education (n = 237, 89%) (Table 1). Two thirds of the cohort were subsistence farmers (n = 175, 66%), which was more common among HIV uninfected participants as compared to PLWH (82% versus 53%, *p* < 0.001). PLWH also tended to be of higher socioeconomic status than HIV uninfected participants (*p* = 0.001). Current smoking was generally uncommon (n = 40, 15%), while biomass exposure in the form of exposure to either firewood or charcoal as cooking fuel was nearly ubiquitous (n = 262, 99%). One fifth of the cohort had a known diagnosis of hypertension, which was more common among HIV uninfected participants as compared to PLWH (26% versus 14%, *p* = 0.02*)*. Few had a known diagnosis of COPD/asthma, diabetes, or hyperlipidemia at study enrollment, though a previous diagnosis of pneumonia or tuberculosis was more common among PLWH as compared to HIV uninfected participants (24% vs 6%, *p* < 0.001). There was some heterogeneity across the four categories of BMI between PLWH and HIV uninfected participants; few participants were underweight (n = 32; 12%), though being underweight was more common among the HIV uninfected study participants (n = 23, 18%) than those living with HIV (n = 9, 6%). Among the 140 PLWH, most were virally suppressed (n = 123, 93%) with evidence of immune reconstitution (n = 83, 82% with CD4 T-cell counts ≥ 350 cells/mm^3^), and had been taking antiretroviral therapy for a median of nine years (interquartile range [IQR] 8 to 10).

**Table 1:** Cohort characteristics

	Total Cohort (*n* = 265)	HIV+ (*n* = 140)	HIV− (*n* = 125)	*p* value
Age, years	52 [49, 56]	52 [49, 55]	53 [49, 56]	0.807
Female sex	125 (47)	65 (46)	60 (48)	0.798
Subsistence farmer	175 (66)	74 (53)	102 (82)	< 0.001
Education				0.138
Did not complete primary school	147 (55)	70 (50)	77 (62)	
Completed primary school	90 (34)	52 (37)	38 (30)	
Completed secondary school	28 (11)	18 (13)	10 (8)	
Asset index (median score per quartile)^†^				0.001
Poorest (− 2.04)	65 (25)	28 (20)	37 (30)	
Poor (− 1.18)	71 (27)	30 (21)	41 (33)	
Less poor (0.23)	64 (24)	35 (25)	29 (23)	
Least poor (2.60)	65 (25)	47 (34)	18 (14)	
Smoking history				0.005
Never Smoker	134 (51)	79 (56)	55 (44)	
Former Smoker	91 (34)	49 (35)	42 (34)	
Current Smoker	40 (15)	12 (9)	28 (22)	
Cooking biomass exposure				< 0.001
Charcoal	37 (14)	35 (25)	2 (2)	
Firewood	225 (85)	102 (73)	123 (98)	
History of pneumonia or TB	40 (15)	33 (24)	7 (6)	< 0.001
Medical comorbidities^††^				
COPD/Asthma	9 (3)	7 (5)	2 (2)	0.179
DM	14 (5)	6 (4)	8 (6)	0.584
HTN	52 (20)	20 (14)	32 (26)	0.021
HL	9 (3)	6 (4)	3 (2)	0.507
Stroke	8 (3)	5 (4)	3 (2)	0.726
MI/CHF	3 (1)	1 (1)	2 (2)	0.603
Total activity per week (min)	9,102 [5,733, 11,664]	7,866 [5238, 10,764]	9743 [6,927, 13,025]	< 0.001
Body mass index (kg/m^3^)				0.012
Underweight (< 18.5)	32 (12)	9 (6)	23 (18)	
Normal (18.5–24.9)	154 (58)	91 (65)	63 (50)	
Overweight (25–29.9)	48 (18)	26 (19)	22 (18)	
Obese (≥ 30)	31 (12)	14 (10)	17 (14)	
HIV Characteristics				
HIV viral load (copies/μL)				
Undetectable		123 (93)		
Detectable, up to 10,000		7 (5)		
> 10,000		2 (2)		
CD4 T-cell count (cells/mm^3^)				
≥ 500		46 (46)		
350–499		37 (37)		
< 350		18 (18)		
ART regimen				
AZT/3TC/NVP or EFV		108 (78)		
TDF/3TC/NVP or EFV		19 (14)		
TDF/3TC/LPV/r		11 (8)		
Tri		1 (1)		
ART duration, years		9 [8, 10]		

(n) % and median [IQR] unless otherwise indicated

Abbreviations: HIV, human immunodeficiency virus; COPD, chronic obstructive pulmonary disease; DM, diabetes mellitus; HTN, hypertension; HL, hyperlipidemia; MI, myocardial infarction; CHF, congestive heart failure; ART, antiretroviral therapy; AZT, zidovudine; NVP, nevirapine; TDF, tenofovir; LPV/r, lopinavir/ritonavir; 3TC, Lamivudine; EFV, Efavirenz; Tri, Triumeq

^†^Asset index total cohort adds up to 101% due to rounding

^††^Medical comorbidities were self-reported

Of the 265 participants, 17 (6%) had COPD by the fixed-ratio spirometric criteria, and the proportion was similar when defining COPD using the lower limit of the expected FEV_1_/FVC ratio. Most of the participants with COPD (n = 13, 76%, *p* = 0.048) were PLWH (Table 2). Median FEV_1_ and FEV_1_/FVC were 2.47 L (IQR 2.07 to 2.95) and 0.80 (IQR 0.77 to 0.84), respectively, and neither differed by HIV serostatus. Median cIMT was 0.67 mm (IQR 0.60 to 0.74), and while PLWH had a slightly lower cIMT than HIV-uninfected participants (PLWH: 0.65 mm [IQR 0.59 to 0.74]; HIV-uninfected: 0.68 mm [IQR 0.62 to 0.74], *p* = 0.076), this difference did not meet statistical significance. There was also no difference in the proportion of people with and without HIV who had a cIMT above the 75^th^ percentile.

**Table 2:** Description of lung function and cardiovascular disease across cohort

Disease measure	Total cohort (n = 265)	HIV+ (n = 140)	HIV− (n = 125)	*p* value*
FEV_1_ (L)	2.47 (2.07, 2.95)	2.40 (2.08, 2.95)	2.52 (2.04, 2.95)	0.853
FVC (L)	3.14 (2.66, 3.70)	3.09 (2.67, 3.76)	3.24 (2.66, 3.68)	0.936
FEV_1_/FVC	0.80 (0.76, 0.83)	0.8 (0.75, 0.83)	0.8 (0.77, 0.83)	0.671
COPD (FEV_1_/FVC < 0.7)	17 (6%)	13 (9%)	4 (3%)	0.044
cIMT (mm)	0.67 (0.6, 0.74)	0.65 (0.59, 0.74)	0.68 (0.62, 0.74)	0.076
cIMT ≥ 75th percentile	67 (25%)	35 (25%)	32 (26%)	0.911

N (%) or median (IQR) unless otherwise indicated

Abbreviations: cIMT, carotid intima media thickness; UGANDAC, Uganda non-communicable diseases and aging cohort; HIV, Human immunodeficiency virus; FEV1, forced expiratory volume in one second; FVC, forced vital capacity in one second; L, liters; mm, millimeters; COPD, chronic obstructive pulmonary disease

^*^Derived from chi2, Fisher’s exact, or Mann–Whitney U (rank sum) statistical tests

In multivariable linear regression models adjusted for age, sex, smoking history, HIV serostatus, and socioeconomic status, lower FEV_1_ was associated with increased cIMT (β = 0.006 per 200 mL FEV_1_ decrease; 95% CI 0.002 to 0.011, *p* = 0.01; Table 3). In pre-specified sub-group analyses (Additional file 1: Tables S3-S8, Figs. S1-S2), there was little evidence of heterogeneity in the relationship between FEV_1_ and cIMT. Although the relationship between FEV_1_ and cIMT was more pronounced among older participants as compared to younger participants (age > 55 β = 0.019, 95% CI 0.008 to 0.030, *p* < 0.01; age ≤ 55 β = 0.004, 95% CI − 0.001 to 0.010, *p* = 0.08), the interaction was not statistically significant. There was heterogeneity in the relationship between FEV_1_ and cIMT between the different strata of socioeconomic status (*p* value for interaction = 0.03), however there was substantial overlap in the point estimates and confidence intervals among the strata and no clear trend that increasing or decreasing wealth modified the relationship. While the increase in cIMT per 200 mL decrease in FEV_1_ appeared to be more pronounced among men as compared to women (β = 0.007, 95% CI 0.001 to 0.013, *p* = 0.02; β = 0.005, 95% CI − 0.004 to 0.014, *p* = 0.24, respectively), those who had ever smoked as compared to never smokers (β = 0.007; 95% CI 0.001 to 0.013, *p* = 0.02; β = 0.006; 95% CI − 0.001 to 0.013, *p* = 0.11, respectively) and HIV uninfected individuals as compared to those with HIV (β 0.009; 95% CI 0.001 to 0.016, *p* = 0.02; β 0.005; 95% CI − 0.001 to 0.011, *p* = 0.1, respectively), none of the interaction terms reached statistical significance (Additional file 1: Table S3).

**Table 3:** Correlates of increased cIMT in the UGANDAC cohort (n = 265)

Characteristic	Unadjusted		Adjusted	
	β (95% CI)	*p* value	β (95% CI)	*p* value
Age (per 5-year increase)	0.031 (0.023, 0.039)	< 0.001	0.028 (0.021, 0.036)	< 0.001
Female sex	0.031 (0.007, 0.054)	0.010	0.012 (− 0.015, 0.039)	0.381
Ever smokers	− 0.003 (− 0.027, 0.020)	0.794	0.004 (− 0.018, 0.026)	0.721
Asset Index				
Poorest	Reference			
Poor	0.015 (− 0.012, 0.041)	0.275	0.037 (0.008, 0.065)	0.012
Less poor	− 0.000 (− 0.028, 0.027)	0.978	0.034 (0.005, 0.064)	0.023
Least poor	0.024 (− 0.003, 0.051)	0.087	0.050 (0.020, 0.081)	0.001
HIV positive	− 0.018 (− 0.041, 0.006)	0.142	− 0.021 (− 0.042, − 0.000)	0.050
FEV_1_ (per 200 mL decrease)	0.010 (0.006, 0.014)	< 0.001	0.006 (0.002, 0.011)	0.007

cIMT, carotid intima media thickness; UGANDAC, Uganda non-communicable diseases and aging cohort; HIV, Human immunodeficiency virus; FEV_1_, forced expiratory volume in one second; mL, milliliters

Reference categories for categorical variables: Sex—male; Ever smokers—lifelong never smokers; Asset index—poorest quartile; HIV Serostatus—HIV negative

Age and FEV_1_ were scaled as reported in the table and centered on median value of the cohort

In sensitivity analyses, the previously observed association between lung function and cIMT was no longer statistically significant when lung function was defined using FEV_1_/FVC rather than FEV_1_ (Additional file 1: Table S9). Including physical activity in the model did not impact the observed relationship between lung function and cIMT (Additional file 1: Table S10).

## Discussion

In summary, we found that impaired lung function was associated with increased cIMT—a measure of cardiovascular disease risk—in a cross-sectional analysis of data from 265 people with and without HIV in southwestern Uganda. There was no substantive evidence to suggest modification of the relationship between FEV_1_ and cIMT across age, sex, smoking history, socioeconomic status or HIV serostatus.

The COPD prevalence in this Ugandan cohort is similar to previously-published COPD prevalence estimates in Uganda that range from 1.5% to 6.1% [50, 51], and to the estimated 6.2% COPD prevalence in the United States [52], but much lower than the estimated 12% COPD prevalence among European populations [53, 54]. Our work expands upon the published literature by suggesting that the relationship between lung dysfunction and cardiovascular disease in high-incomes settings [3, 55] is also present in a sub-Saharan African population. Demonstrating this relationship in southwestern Uganda highlights the need for future research that seeks to identify risk-factors for co-occurring cardiopulmonary disease in the region and develop scalable strategies for preventing and treating multimorbidity [56]. This is particularly important in sub-Saharan Africa where the etiology of chronic lung and cardiovascular disease may differ from high-income settings, as the rates of smoking are lower, exposure to air pollution is higher, and there is a greater burden of HIV and other infections.

We found that a lung function decrement of 200 mL was associated with 0.006 mm greater cIMT. A recent meta-analysis of 119 randomized clinical trials involving a total of 100,667 participants found that an increase in cIMT by little as 0.01 mm per year over an average follow up time of 3.7 years led to a clinically significant change (relative risk of 0.91) in the risk of myocardial infarction, stroke, revascularization or fatal cardiovascular event [42]. This suggests that the results of our work highlight a clinically significant relationship that may guide clinicians in identifying comorbid lung and cardiovascular disease. The lack of a relationship between FEV_1_/FVC and cIMT could have two potential sources. The seminal papers on the development of COPD among smokers highlights that FEV_1_ decline precedes the development of obstructive lung disease per se [57], suggesting that FEV_1_ is a more sensitive marker of early lung dysfunction. Alternatively, FEV_1_ decline in the absence of FEV_1_/FVC decline suggests that the underlying lung dysfunction results from restrictive rather than obstructive lung disease. We do not have lung volume measurements in this cohort and so cannot formally evaluate the relationship between decrements in total lung capacity (the lung function parameter required for diagnosis of restrictive lung disease) and cIMT.

Our exploratory subgroup analyses identified potential variations in the relationship between FEV_1_ and cIMT. Contrary to data from high income settings [58], there was no clear trend that low socioeconomic status was associated with the largest increase in pre-clinical atherosclerosis for a given decrement in lung function. Prior work from Garin et al., showed that the way in which socioeconomic status influenced the odds of multimorbidity differed in high-income as compared to low-income countries [58]. Among participants from high-income countries, the odds of multimorbidity were higher among those with lower socioeconomic status. However, in low-income countries, the odds of multimorbidity were higher among those with higher socioeconomic status. While much work has focused on relationships between wealth-related factors and non-communicable diseases like diabetes and cardiovascular disease, few have focused on pulmonary disease [59]. Multimorbidity risk factors like diet, activity, and smoking behaviors vary across socioeconomic strata, but also vary by country. For example, behaviors such as smoking and unhealthy dietary choices are more prevalent among lower socioeconomic strata within higher-income countries, while smoking and unhealthy dietary choices are more prevalent among higher socioeconomic strata in lower-income countries [60, 61]. Exposure to air pollutants also varies based on socioeconomic status in ways that are not uniform across regions and countries [62]. These differences in risk factor epidemiology and the ways in which they may influence concomitant cardiopulmonary disease risk in sub-Saharan Africa warrant further investigation.

The association between lung function and pre-clinical atherosclerosis was more than four times stronger among participants at least 55 years of age as compared to participants 55 years of age and younger, though there was no evidence of effect measure modification and there was significant overlap in 95% confidence intervals for the effect estimates between both age groups. However the observed trend is similar to those described in a multinational, population-based study of multimorbidity [58]. Among nearly 42,000 individuals of at least 50 years of age from nine countries, multimorbidity (defined as two or more chronic conditions) was also more likely among older individuals as compared to younger individuals. COPD and cardiovascular disease share several risk factors associated with persistent systemic inflammation [9–11], including smoking, air pollution, and infection. It is possible that as the body and immune system ages, chronic inflammation may lead to more cardiopulmonary damage [63, 64] and the stronger observed relationship between impaired lung function and cardiovascular disease in this cohort.

Interestingly, we found that the association between lung function and pre-clinical cardiovascular disease was similar among men and women, despite reported differences in indoor air pollution exposure among women due to gender-based cooking roles in the region [65–67]. This may suggest that, while women may have higher risk of lung dysfunction related to biomass exposure, that the associated risk of pre-clinical atherosclerosis among those with impaired lung function may not differ based on sex. Our ongoing work in this cohort to quantify air pollution exposure and follow indices of lung and cardiovascular disease over time will hopefully shed light on these complex relationships between air pollution, sex, and cardiopulmonary disease.

We found no evidence that HIV serostatus modified the relationship between impaired lung function and pre-clinical atherosclerosis. In fact, in models stratified by HIV serostatus, the increase in cIMT per 200 mL decrease in FEV_1_ was nearly twice as large among HIV uninfected participants as compared to PLWH, though the 95% confidence intervals for the effect estimates in both PLWH and HIV-uninfected comparators were largely overlapping. Though we may have been underpowered to detect effect modification by HIV serostatus, these data suggest that treated HIV infection may not be an independent risk factor for concomitant lung and cardiovascular disease. It is plausible that this finding may be explained by survivor bias, as participants in this cross-sectional cohort had to have lived to at least 40 years of age to be eligible for study participation. While both PLWH and HIV uninfected comparators would have to be healthy enough to live to 40 years of age, PLWH globally have a shorter life expectancy [29], so those living into adulthood may have been disproportionately the healthiest sub-population of PLWH [68]. To definitively characterize the influence of HIV serostatus on the risk of concurrent cardiopulmonary disease, we are following this population of adults with and without HIV through adulthood to compare differences in lung and cardiovascular disease prevalence over time.

Globally, smoking is the leading cause of both lung and cardiovascular disease [69]. However, in our analysis, the relationship between FEV_1_ and cIMT persisted despite controlling for smoking, which suggests that non-tobacco shared risk factors might contribute to both cardiovascular and pulmonary disease in the region. The African continent is home to some of the highest ambient air pollution concentrations in the world, driven largely by rapid urbanization, incomplete vehicle and industry emissions regulations, and the use of biomass fuels [70, 71]. Particulate matter causes both alveolar and systemic inflammation [72–75] that is associated with increased risk of both cardiovascular disease and acute coronary events [11, 76–80]. Using ambulatory air quality monitors, our group has demonstrated that personal air pollution exposure in southwestern Uganda is higher than international air quality standards and associated with respiratory morbidity [81]. Our ongoing work—which includes longitudinal measures of air pollution exposure, lung function, alveolar inflammation, and cardiovascular health—is designed to explore the causality and clinical implications of these complex relationships. As global smoking prevalence continues to decline [12, 14] and rapid industrialization and urbanization continue [82], air pollution is poised to replace tobacco as a leading global cause of cardiopulmonary disease, so understanding these multifaceted relationships is crucial to improve health outcomes.

The main strength of this analysis is that it is based within a well-designed cohort study of people with and without HIV that leverages carefully collected biologic measures of lung function and cardiovascular disease rather than relying upon self-reported metrics [83]. There are also several limitations. As a cross-sectional study, we can make no inferences as to the causality of the demonstrated relationships between lung function and pre-clinical atherosclerosis. Additionally, tobacco use—an important driver of both lung and cardiovascular disease—was categorized via self-report and therefore subject to misclassification bias. Moreover, nearly all PLWH had evidence of immune reconstitution, so the relationships demonstrated in this analysis may not be reflective of people with undiagnosed or untreated HIV. However, advances in antiretroviral therapy access and initiation have led to better viral control and longer life expectancy [29], so the population of PLWH exhibiting viral control is the population for whom the relationships between co-occurring chronic diseases is crucial to understand. Furthermore, air pollution is an important regional exposure and was classified through self-report rather than air quality measurements, so we could not quantify differences in exposure magnitude. However, the ubiquitous self-report of biomass exposure is consistent with the body of literature on biomass exposure in sub-Saharan Africa [70], and thus our self-reported data are likely to grossly reflect objective exposure metrics. Dietary patterns have been associated with the development of lung and cardiovascular disease and could thus be the source of residual confounding in our models. Additionally, our small sample size limits our power to definitively characterize the potential effect modification by HIV serostatus and other risk factors, though our stratified analyses provide important hypothesis-generating findings that will inform future longitudinal studies of larger study populations. We characterize socioeconomic status using quartiles of asset ownership index among the study population—a common practice in resource-limited settings, but one that may limit the ability to compare our findings related to socioeconomic status to other published literature. Finally, as is true for all observational cohort studies, our results may be susceptible to unmeasured and residual confounding. Despite these limitations, our findings are generalizable to adults with and without HIV who are living in similar rural and semi-urban settings in Uganda and East Africa.

## Conclusions

In conclusion, our work corroborates the presence of concomitant cardiovascular disease among the estimated 26 million people living with chronic lung disease in sub-Saharan Africa. Characterizing the prevalence and shared risk factors of co-occurring chronic diseases in sub-Saharan Africa—where most of the upcoming population growth is projected to occur [84]—is crucial to expand our understanding of the epidemiology of non-communicable disease among at-risk populations. Further work is necessary to characterize how concomitant non-communicable diseases influence health outcomes in sub-Saharan Africa, and how to effectively leverage the existing long-term care infrastructure—established largely through decades of concerted international cooperation to combat the HIV epidemic [85]—to improve prevention, detection and management of chronic non-communicable disease in the most cost-effective manner.

## Supplementary Information

**Additional file 1**. Data Supplement.

## Data Availability

The datasets generated and/or analysed during the current study are available from the corresponding author on reasonable request.

## Abbreviations

ATS: American Thoracic Society
cIMT: Carotid intima media thickness
COPD: Chronic obstructive pulmonary disease
FEV_1_: Forced expiratory volume in one second
FVC: Forced vital capacity
GOLD: Global Initiative for Obstructive Lung Disease
HIV: Human immunodeficiency virus
IQR: Interquartile range
mL: Milliliters
µL: Microliters
NHANES: National Health and Nutrition Examination Survey
PLWH: People living with HIV
UGANDAC: Uganda Non-Communicable Diseases and Aging Cohort
WHO: STEPS: World Health Organization STEPwise approach to surveillance

## References

[1] Roth GA, Abate D, Abate KH, Abay SM, Abbafati C, Abbasi N (2018). Global, regional, and national age-sex-specific mortality for 282 causes of death in 195 countries and territories, 1980–2017: a systematic analysis for the Global Burden of Disease Study 2017. Lancet.

[2] Roversi S, Roversi P, Spadafora G, Rossi R, Fabbri LM (2014). Coronary artery disease concomitant with chronic obstructive pulmonary disease. Eur J Clin Invest.

[3] Chen W, Thomas J, Sadatsafavi M, FitzGerald JM (2015). Risk of cardiovascular comorbidity in patients with chronic obstructive pulmonary disease: a systematic review and meta-analysis. Lancet Respir Med.

[4] Nishiyama K, Morimoto T, Furukawa Y, Nakagawa Y, Ehara N, Taniguchi R (2010). Chronic obstructive pulmonary disease–an independent risk factor for long-term cardiac and cardiovascular mortality in patients with ischemic heart disease. Int J Cardiol.

[5] Dalal AA, Shah M, Lunacsek O, Hanania NA (2011). Clinical and economic burden of patients diagnosed with COPD with comorbid cardiovascular disease. Respir Med.

[6] Sin DD, Wu L, Man SF (2005). The relationship between reduced lung function and cardiovascular mortality: a population-based study and a systematic review of the literature. Chest.

[7] Adeloye D, Basquill C, Papana A, Chan KY, Rudan I, Campbell H (2015). An estimate of the prevalence of COPD in Africa: a systematic analysis. COPD.

[8] Mensah GA, Roth GA, Sampson UKA, Moran AE, Feigin VL, Forouzanfar MH (2015). Mortality from cardiovascular diseases in sub-Saharan Africa, 1990–2013: a systematic analysis of data from the Global Burden of Disease Study 2013. Cardiovasc J Afr.

[9] Hole DJ, Watt GC, Davey-Smith G, Hart CL, Gillis CR, Hawthorne VM. Impaired lung function and mortality risk in men and women: findings from the Renfrew and Paisley prospective population study. BMJ. 1996;313(7059):711–5; discussion 5–6. 10.1136/bmj.313.7059.711 PMC2352103 8819439

[10] Thomsen M, Dahl M, Lange P, Vestbo J, Nordestgaard BG (2012). Inflammatory biomarkers and comorbidities in chronic obstructive pulmonary disease. Am J Respir Crit Care Med.

[11] Van Eeden S, Leipsic J, Paul Man SF, Sin DD (2012). The relationship between lung inflammation and cardiovascular disease. Am J Respir Crit Care Med.

[12] Reitsma MBFN, Ng M (2017). Smoking prevalence and attributable disease burden in 195 countries and territories, 1990–2015: a systematic analysis from the Global Burden of Disease Study 2015. Lancet.

[13] Brathwaite R, Addo J, Smeeth L, Lock K (2015). A systematic review of tobacco smoking prevalence and description of tobacco control strategies in sub-Saharan African countries; 2007 to 2014. PLoS ONE.

[14] WHO global report on trends in prevalence of tobacco smoking 2000 - 2025, second edition. Geneva: World Health Organization; 2018.

[15] Cohen AJ, Brauer M, Burnett R, Anderson HR, Frostad J, Estep K (2017). Estimates and 25-year trends of the global burden of disease attributable to ambient air pollution: an analysis of data from the Global Burden of Diseases Study 2015. Lancet.

[16] Institute HE. State of Global Air 2019. Accessed [March 9, 2021], at www.stateofglobalair.org.

[17] Alexeeff SE, Deosaransingh K, Liao NS, Van Den Eeden SK, Schwartz J, Sidney S. Particulate matter and cardiovascular risk in adults with chronic obstructive pulmonary disease. Am J Respir Crit Care Med 2021. 10.1164/rccm.202007-2901OC PMC8650791 33662228

[18] UNAIDS. Global HIV & AIDS statistics - 2020 fact sheet. Accessed [March 9, 2021] at https://www.unaids.org/en/resources/fact-sheet.

[19] Crothers K, Butt AA, Gibert CL, Rodriguez-Barradas MC, Crystal S, Justice AC (2006). Increased COPD among HIV-positive compared to HIV-negative veterans. Chest.

[20] Zanni MV, Schouten J, Grinspoon SK, Reiss P (2014). Risk of coronary heart disease in patients with HIV infection. Nat Rev Cardiol.

[21] North CM, Muyanja D, Kakuhikire B, Tsai AC, Tracy RP, Hunt PW (2018). Brief report: systemic inflammation, immune activation, and impaired lung function among people living with HIV in rural Uganda. J Acquir Immune Defic Syndr.

[22] Akanbi MO, Taiwo BO, Achenbach CJ, Ozoh OB, Obaseki DO, Sule H, et al. HIV associated chronic obstructive pulmonary disease in Nigeria. J AIDS Clin Res. 2015;6(5). 10.4172/2155-6113.1000453 PMC4521629 26236557

[23] Pefura-Yone EW, Fodjeu G, Kengne AP, Roche N, Kuaban C (2015). Prevalence and determinants of chronic obstructive pulmonary disease in HIV infected patients in an African country with low level of tobacco smoking. Respir Med.

[24] Bigna JJ, Kenne AM, Asangbeh SL, Sibetcheu AT (2018). Prevalence of chronic obstructive pulmonary disease in the global population with HIV: a systematic review and meta-analysis. Lancet Glob Health.

[25] Muiru AN, Bibangambah P, Hemphill L, Sentongo R, Kim JH, Triant VA (2018). Distribution and performance of cardiovascular risk scores in a mixed population of HIV-infected and community-based HIV-uninfected individuals in Uganda. J Acquir Immune Defic Syndr.

[26] Nonterah EA, Boua PR, Klipstein-Grobusch K, Asiki G, Micklesfield LK, Agongo G (2019). Classical cardiovascular risk factors and HIV are associated with carotid intima-media thickness in adults from sub-Saharan Africa: findings from H3Africa AWI-Gen study. J Am Heart Assoc.

[27] Wang H, Abbas KM, Abbasifard M, Abbasi-Kangevari M, Abbastabar H, Abd-Allah F (2020). Global age-sex-specific fertility, mortality, healthy life expectancy (HALE), and population estimates in 204 countries and territories, 1950–2019: a comprehensive demographic analysis for the Global Burden of Disease Study 2019. Lancet.

[28] Bor J, Herbst AJ, Newell M-L, Bärnighausen T (2013). Increases in adult life expectancy in rural south africa: valuing the scale-up of HIV treatment. Science.

[29] Wandeler G, Johnson LF, Egger M (2016). Trends in life expectancy of HIV-positive adults on antiretroviral therapy across the globe: comparisons with general population. Curr Opin HIV AIDS.

[30] Gouda HN, Charlson F, Sorsdahl K, Ahmadzada S, Ferrari AJ, Erskine H (2019). Burden of non-communicable diseases in sub-Saharan Africa, 1990–2017: results from the Global Burden of Disease Study 2017. Lancet Glob Health.

[31] Smit M, Brinkman K, Geerlings S, Smit C, Thyagarajan K, van Sighem A (2015). Future challenges for clinical care of an ageing population infected with HIV: a modelling study. Lancet Infect Dis.

[32] Smith CJ, Ryom L, Weber R, Morlat P, Pradier C, Reiss P (2014). Trends in underlying causes of death in people with HIV from 1999 to 2011 (D:A:D): a multicohort collaboration. Lancet.

[33] Katende D, Mutungi G, Baisley K, Biraro S, Ikoona E, Peck R (2015). Readiness of Ugandan health services for the management of outpatients with chronic diseases. Trop Med Int Health.

[34] Settumba SN, Sweeney S, Seeley J, Biraro S, Mutungi G, Munderi P (2015). The health system burden of chronic disease care: an estimation of provider costs of selected chronic diseases in Uganda. Trop Med Int Health.

[35] Jaffar S, Amberbir A, Kayuni N, Musicha C, Nyirenda M (2013). Viewpoint: scaling up testing services for non-communicable diseases in Africa: priorities for implementation research. Trop Med Int Health.

[36] North CM, Allen JG, Okello S, Sentongo R, Kakuhikire B, Ryan ET (2018). HIV infection, pulmonary tuberculosis, and COPD in rural Uganda: a cross-sectional study. Lung.

[37] Siedner MJ, Kim JH, Nakku RS, Hemphill L, Triant VA, Haberer JE (2016). HIV infection and arterial stiffness among older-adults taking antiretroviral therapy in rural Uganda. AIDS.

[38] Takada S, Nyakato V, Nishi A, O'Malley AJ, Kakuhikire B, Perkins JM (2019). The social network context of HIV stigma: Population-based, sociocentric network study in rural Uganda. Soc Sci Med.

[39] Filmer D, Pritchett LH (2001). Estimating wealth effects without expenditure data—or tears: an application to educational enrollments in states of India. Demography.

[40] Smith ML, Kakuhikire B, Baguma C, Rasmussen JD, Bangsberg DR, Tsai AC (2020). Do household asset wealth measurements depend on who is surveyed? Asset reporting concordance within multi-adult households in rural Uganda. J Glob Health.

[41] WHO. The STEPS Instrument and Support Materials. [cited August 6, 2020. Available from: http://www.who.int/chp/steps/riskfactor/en/.

[42] Willeit P, Tschiderer L, Allara E, Reuber K, Seekircher L, Gao L (2020). Carotid Intima-Media Thickness Progression as Surrogate Marker for Cardiovascular Risk. Circulation.

[43] Stein JH, Korcarz CE, Hurst RT, Lonn E, Kendall CB, Mohler ER (2008). Use of carotid ultrasound to identify subclinical vascular disease and evaluate cardiovascular disease risk: a consensus statement from the American Society of Echocardiography Carotid Intima-Media thickness task force endorsed by the society for vascular medicine. J Am Soc Echocardiogr.

[44] Miller MR, Hankinson J, Brusasco V, Burgos F, Casaburi R, Coates A (2005). Standardisation of spirometry. Eur Respir J.

[45] Pellegrino R, Viegi G, Brusasco V, Crapo RO, Burgos F, Casaburi R (2005). Interpretative strategies for lung function tests. Eur Respir J.

[46] Hankinson JL, Odencrantz JR, Fedan KB (1999). Spirometric reference values from a sample of the general U.S. population. Am J Respir Crit Care Med.

[47] Vestbo J, Hurd SS, Agusti AG, Jones PW, Vogelmeier C, Anzueto A (2013). Global strategy for the diagnosis, management, and prevention of chronic obstructive pulmonary disease: GOLD executive summary. Am J Respir Crit Care Med.

[48] Ssinabulya I, Kayima J, Longenecker C, Luwedde M, Semitala F, Kambugu A (2014). Subclinical atherosclerosis among HIV-infected adults attending HIV/AIDS care at two large ambulatory HIV clinics in Uganda. PLoS ONE.

[49] Stein JH, Korcarz CE, Hurst RT, Lonn E, Kendall CB, Mohler ER, et al. Use of carotid ultrasound to identify subclinical vascular disease and evaluate cardiovascular disease risk: a consensus statement from the American Society of Echocardiography Carotid Intima-Media Thickness Task Force. Endorsed by the Society for Vascular Medicine. J Am Soc Echocardiogr. 2008;21(2):93–111; quiz 89–90. 10.1016/j.echo.2007.11.011 18261694

[50] North CM, Kakuhikire B, Vorechovska D, Hausammann-Kigozi S, McDonough AQ, Downey J (2019). Prevalence and correlates of chronic obstructive pulmonary disease and chronic respiratory symptoms in rural southwestern Uganda: a cross-sectional, population-based study. J Glob Health.

[51] Siddharthan T, Grigsby M, Morgan B, Kalyesubula R, Wise RA, Kirenga B (2019). Prevalence of chronic respiratory disease in urban and rural Uganda. Bull World Health Organ.

[52] Wheaton AG, Liu Y, Croft JB, VanFrank B, Croxton TL, Punturieri A (2019). Chronic obstructive pulmonary disease and smoking status—United States, 2017. MMWR Morb Mortal Wkly Rep.

[53] Blanco I, Diego I, Bueno P, Fernandez E, Casas-Maldonado F, Esquinas C (2018). Geographical distribution of COPD prevalence in Europe, estimated by an inverse distance weighting interpolation technique. Int J Chron Obstruct Pulmon Dis.

[54] Adeloye D, Chua S, Lee C, Basquill C, Papana A, Theodoratou E (2015). Global and regional estimates of COPD prevalence: systematic review and meta-analysis. J Glob Health.

[55] Mullerova H, Agusti A, Erqou S, Mapel DW (2013). Cardiovascular comorbidity in COPD: systematic literature review. Chest.

[56] Adeloye D, Agarwal D, Barnes PJ, Bonay M, van Boven JF, Bryant J (2021). Research priorities to address the global burden of chronic obstructive pulmonary disease (COPD) in the next decade. J Glob Health.

[57] Fletcher C, Peto R (1977). The natural history of chronic airflow obstruction. BMJ.

[58] Garin N, Koyanagi A, Chatterji S, Tyrovolas S, Olaya B, Leonardi M (2015). Global multimorbidity patterns: a cross-sectional, population-based, multi-country study. J Gerontol Ser A.

[59] Williams J, Allen L, Wickramasinghe K, Mikkelsen B, Roberts N, Townsend N (2018). A systematic review of associations between non-communicable diseases and socioeconomic status within low- and lower-middle-income countries. J Glob Health.

[60] Jones-Smith JC, Gordon-Larsen P, Siddiqi A, Popkin BM (2012). Is the burden of overweight shifting to the poor across the globe? Time trends among women in 39 low-and middle-income countries (1991–2008). Int J Obes.

[61] Jones-Smith JC, Gordon-Larsen P, Siddiqi A, Popkin BM (2011). Cross-national comparisons of time trends in overweight inequality by socioeconomic status among women using repeated cross-sectional surveys from 37 developing countries, 1989–2007. Am J Epidemiol.

[62] Hajat A, Hsia C, O’Neill MS (2015). Socioeconomic disparities and air pollution exposure: a global review. Curr Environ Health Rep.

[63] North BJ, Sinclair DA (2012). The intersection between aging and cardiovascular disease. Circ Res.

[64] Provinciali M, Cardelli M, Marchegiani F (2011). Inflammation, chronic obstructive pulmonary disease and aging. Curr Opin Pulm Med.

[65] van Gemert F, van der Molen T, Jones R, Chavannes N (2011). The impact of asthma and COPD in sub-Saharan Africa. Prim Care Respir J.

[66] Perez-Padilla R, Schilmann A, Riojas-Rodriguez H (2010). Respiratory health effects of indoor air pollution. Int J Tuberc Lung Dis.

[67] Po JY, FitzGerald JM, Carlsten C (2011). Respiratory disease associated with solid biomass fuel exposure in rural women and children: systematic review and meta-analysis. Thorax.

[68] Manne-Goehler J, Montana L, Gomez-Olive FX, Rohr J, Harling G, Wagner RG (2017). The ART advantage: health care utilization for diabetes and hypertension in rural South Africa. J Acquir Immune Defic Syndr.

[69] GBD Risk Factors Collaborators (2017). Global, regional, and national comparative risk assessment of 84 behavioural, environmental and occupational, and metabolic risks or clusters of risks, 1990–2016: a systematic analysis for the Global Burden of Disease Study 2016. Lancet.

[70] Karagulian F, Belis CA, Dora CFC, Prüss-Ustün AM, Bonjour S, Adair-Rohani H (2015). Contributions to cities' ambient particulate matter (PM): a systematic review of local source contributions at global level. Atmos Environ.

[71] Brauer M, Freedman G, Frostad J, van Donkelaar A, Martin RV, Dentener F (2016). Ambient air pollution exposure estimation for the global burden of disease 2013. Environ Sci Technol.

[72] Ghio AJ, Devlin RB (2001). Inflammatory lung injury after bronchial instillation of air pollution particles. Am J Respir Crit Care Med.

[73] Becker S, Soukup JM, Sioutas C, Cassee FR (2003). Response of human alveolar macrophages to ultrafine, fine, and coarse urban air pollution particles. Exp Lung Res.

[74] Rylance J, Fullerton DG, Scriven J, Aljurayyan AN, Mzinza D, Barrett S (2015). Household air pollution causes dose-dependent inflammation and altered phagocytosis in human macrophages. Am J Respir Cell Mol Biol.

[75] Kido T, Tamagawa E, Bai N, Suda K, Yang HH, Li Y (2011). Particulate matter induces translocation of IL-6 from the lung to the systemic circulation. Am J Respir Cell Mol Biol.

[76] Hajat A, Allison M, Diez-Roux AV, Jenny NS, Jorgensen NW, Szpiro AA (2015). Long-term exposure to air pollution and markers of inflammation, coagulation, and endothelial activation: a repeat-measures analysis in the Multi-Ethnic Study of Atherosclerosis (MESA). Epidemiology.

[77] Hatzis C, Godleski JJ, Gonzalez-Flecha B, Wolfson JM, Koutrakis P (2006). Ambient particulate matter exhibits direct inhibitory effects on oxidative stress enzymes. Environ Sci Technol.

[78] Pope CA, Bhatnagar A, McCracken JP, Abplanalp W, Conklin DJ, O'Toole T (2016). Exposure to fine particulate air pollution is associated with endothelial injury and systemic inflammation. Circ Res.

[79] Chuang KJ, Chan CC, Su TC, Lee CT, Tang CS (2007). The effect of urban air pollution on inflammation, oxidative stress, coagulation, and autonomic dysfunction in young adults. Am J Respir Crit Care Med.

[80] Mutlu GM, Green D, Bellmeyer A, Baker CM, Burgess Z, Rajamannan N (2007). Ambient particulate matter accelerates coagulation via an IL-6-dependent pathway. J Clin Invest.

[81] North CM, MacNaughton P, Lai PS, Vallarino J, Okello S, Kakuhikire B (2019). Personal carbon monoxide exposure, respiratory symptoms, and the potentially modifying roles of sex and HIV infection in rural Uganda: a cohort study. Environ Health.

[82] Han L, Zhou W, Li W, Li L (2014). Impact of urbanization level on urban air quality: a case of fine particles (PM2.5) in Chinese cities. Environ Pollut.

[83] Mehrotra A, Akanbi MO, Gordon SB (2009). The burden of COPD in Africa: a literature review and prospective survey of the availability of spirometry for COPD diagnosis in Africa. Trop Med Int Health.

[84] Vollset SE, Goren E, Yuan CW, Cao J, Smith AE, Hsiao T, et al. Fertility, mortality, migration, and population scenarios for 195 countries and territories from 2017 to 2100: a forecasting analysis for the Global Burden of Disease Study. Lancet. 2020. 10.1016/S0140-6736(20)30677-2 PMC7561721 32679112

[85] El-Sadr WM, Holmes CB, Mugyenyi P, Thirumurthy H, Ellerbrock T, Ferris R (2012). Scale-up of HIV treatment through PEPFAR: a historic public health achievement. J Acquir Immune Defic Syndr.

