# Supplementary material for: Lung function and atherosclerosis: a cross-sectional study of multimorbidity in rural Uganda
Source: BMC Pulm Med. 2022 Jan 5;22:12. doi: 10.1186/s12890-021-01792-0 (PMC8728924; doi:10.1186/s12890-021-01792-0)
Supplement: Supplementary file 1 — Additional file 1. Data Supplement. [file 12890_2021_1792_MOESM1_ESM.docx]

**Data Supplement**

**Table S1:** Cohort characteristics of those with as compared to those without concomitant spirometry and cIMT measurements

|  | **Concomitant PFT & cIMT measurements**  **(*n*=277)** | **Without concomitant PFT & cIMT**  **(*n*=11)** | ***p* value** |
| --- | --- | --- | --- |
| Age, years | 52 [49,56] | 55 [49,71] | 0.271 |
| HIV positive | 144 (52) | 4 (36) | 0.367 |
| Female sex | 129 (47) | 6 (55) | 0.760 |
| Subsistence farmer | 183 (66) | 8 (73) | 0.756 |
| Education^†^ |  |  | 0.903 |
| Did not complete primary school | 154 (56) | 7 (64) |  |
| Completed primary school | 93 (34) | 3 (27) |  |
| Completed secondary school | 30 (11) | 1 (9) |  |
| Asset index^††^ |  |  | 0.851 |
| Poorest | 67 (24) | 4 (36) |  |
| Poor | 73 (26) | 3 (27) |  |
| Less poor | 69 (25) | 2 (18) |  |
| Least poor | 68 (25) | 2 (18) |  |
| Smoking history |  |  | 0.198 |
| Never Smoker | 141 (51) | 8 (73) |  |
| Former Smoker | 95 (34) | 1 (9) |  |
| Current Smoker | 41 (15) | 2 (18) |  |
| Cooking biomass exposure |  |  | 1.000 |
| Charcoal | 40 (14) | 1 (9) |  |
| Firewood | 235 (85) | 10 (91) |  |
| History of pneumonia or TB | 38 (14) | 3 (27) | 0.195 |
| Medical comorbidities^†††^ |  |  |  |
| COPD/Asthma | 8 (3) | 0 (0) | 1.000 |
| DM | 16 (6) | 0 (0) | 1.000 |
| HTN | 54 (19) | 5 (45) | 0.052 |
| HDL | 10 (4) | 1 (9) | 0.353 |
| Stroke | 10 (4) | 0 (0) | 1.000 |
| MI/CHF | 4 (1) | 0 (0) | 1.000 |
| Body mass index (kg/m^3^) |  |  | 0.458 |
| Underweight (<18.5) | 34 (12) | 1 (9) |  |
| Normal (18.5-24.9) | 160 (58) | 6 (55) |  |
| Overweight (25-29.9) | 51 (18) | 1 (9) |  |
| Obese (≥30) | 32 (12) | 3 (27) |  |
| HIV Characteristics |  |  |  |
| HIV viral load (copies/μL)^††††^ |  |  | 1.000 |
| Undetectable | 127 (94) | 4 (100) |  |
| Detectable, up to 10,000 | 6 (4) | 0 (0) |  |
| >10,000 | 2 (1) | 0 (0) |  |
| CD4 T-cell count (cells/mm^3^) |  |  | 0.085 |
| ≥ 500 | 48 (45) | 1 (33) |  |
| 350-499 | 41 (38) | 0 (0) |  |
| <350 | 18 (17) | 2 (67) |  |
| ART regimen |  |  | 1.000 |
| AZT/3TC/NVP or EFV | 112 (78) | 4 (100) |  |
| TDF/3TC/NVP or EFV | 19 (13) | 0 (0) |  |
| TDF/3TC/LPV/r | 12 (8) | 0 (0) |  |
| Tri | 1 (1) | 0 (0) |  |
| ART duration, years | 9 [8,10] | 9 [9,10] | 0.253 |
| **Table S1 Legend**  n (%) and median [IQR] unless otherwise indicated  Abbreviations: PFT, pulmonary function test; cIMT, carotid intima media thickness; HIV, human immunodeficiency virus; COPD, chronic obstructive pulmonary disease; DM, diabetes mellitus; HTN, hypertension; HDL, High Cholesterol; MI, myocardial infarction; CHF, congestive heart failure; ART, antiretroviral therapy; AZT, zidovudine; NVP, nevirapine; TDF, tenofovir; LPV/r, lopinavir/ritonavir; 3TC, Lamivudine; EFV, Efavirenz, or Tri, triumeq  ^†^Education concomitant PFT & cIMT measurements adds up to 101% due to rounding  ^††^Asset index without concomitant PFT & cIMT adds up to 99% due to rounding  ^†††^Medical comorbidities were self-reported  ^††††^HIV viral load concomitant PFT & cIMT measurements adds up to 99% due to rounding | | | |

**Table S2:** Cohort characteristics of those with concomitant spirometry and cIMT measurements (n=277) who completed ATS-acceptable spirometry as compared to ATS-unacceptable spirometry

|  | **ATS-acceptable spirometry**  **(*n*=265)** | **ATS-unacceptable spirometry**  **(*n*=12)** | ***p* value** |
| --- | --- | --- | --- |
| Age, years | 52 [49,56] | 51 [48,56] | 0.441 |
| Female sex | 125 (47) | 4 (33) | 0.391 |
| Subsistence farmer | 176 (66) | 8 (67) | 1.000 |
| Education |  |  | 0.686 |
| Did not complete primary school | 147 (55) | 7 (58) |  |
| Completed primary school | 90 (34) | 5 (42) |  |
| Completed secondary school | 28 (11) | 0 (0) |  |
| Asset index |  |  | 1.000 |
| Poorest | 65 (25) | 3 (25) |  |
| Poor | 71 (27) | 3 (25) |  |
| Less Poor | 64 (24) | 3 (25) |  |
| Least Poor | 65 (25) | 3 (25) |  |
| Smoking history^†^ |  |  | 0.924 |
| Never Smoker | 134 (51) | 7 (58) |  |
| Former Smoker | 91 (34) | 4 (33) |  |
| Current Smoker | 40 (15) | 1 (8) |  |
| Cooking biomass exposure |  |  | 0.468 |
| Charcoal | 37 (14) | 3 (25) |  |
| Firewood | 225 (85) | 9 (75) |  |
| History of pneumonia or TB | 40 (15) | 0 (0) | 0.225 |
| Medical comorbidities^††^ |  |  |  |
| COPD/Asthma | 9 (3) | 0 (0) | 1.000 |
| DM | 14 (5) | 2 (17) | 0.147 |
| HTN | 52 (20) | 3 (25) | 0.710 |
| HDL | 9 (3) | 1 (8) | 0.363 |
| Stroke | 8 (3) | 0 (0) | 1.000 |
| MI/CHF | 3 (1) | 0 (0) | 1.000 |
| Body mass index (kg/m^3^) |  |  | 0.60 |
| Underweight (<18.5) | 32 (12) | 3 (25) |  |
| Normal (18.5-24.9) | 154 (58) | 6 (50) |  |
| Overweight (25-29.9) | 48 (18) | 2 (17) |  |
| Obese (≥30) | 31 (12) | 1 (8) |  |
| HIV positive | 140 (53) | 4 (33) | 0.242 |
| HIV Characteristics |  |  |  |
| HIV viral load (copies/μL) |  |  | 1.000 |
| Undetectable | 123 (93) | 4 (100) |  |
| Detectable, up to 10,000 | 7 (5) | 0 (0) |  |
| >10,000 | 2 (2) | 0 (0) |  |
| CD4 T-cell count (cells/mm^3^) ^†††^ |  |  | 0.549 |
| ≥ 500 | 46 (46) | 0 (0) |  |
| 350-499 | 37 (37) | 1 (100) |  |
| <350 | 18 (18) | 0 (0) |  |
| ART regimen ^††††^ |  |  | 1.000 |
| AZT/3TC/NVP or EFV | 108 (78) | 4 (100) |  |
| TDF/3TC/NVP or EFV | 19 (14) | 0 (0) |  |
| TDF/3TC/LPV/r | 11 (8) | 0 (0) |  |
| Tri | 1 (1) | 0 (0) |  |
| ART duration, years | 9 [8,10] | 9 [8,9] | 0.477 |
| **Table S2 Legend**  n (%) and median [IQR] unless otherwise indicated  Abbreviations: cIMT, carotid intima media thickness; ATS, American Thoracic Society; HIV, human immunodeficiency virus; COPD, chronic obstructive pulmonary disease; DM, diabetes mellitus; HTN, hypertension; HDL, High Cholesterol; MI, myocardial infarction; CHF, congestive heart failure; ART, antiretroviral therapy; AZT, zidovudine; NVP, nevirapine; TDF, tenofovir; LPV/r, lopinavir/ritonavir; 3TC, Lamivudine; EFV, Efavirenz, or Tri, triumeq  ^†^Smoking history ATS-unacceptable spirometry adds up to 99% due to rounding  ^††^Medical comorbidities were self-reported  ^†††^CD4 T-cell count ATS-acceptable spirometry adds up to 101% due to rounding  ^††††^ ART regimen ATS-acceptable spirometry adds up to 101% due to rounding | | | |

**Table S3:** Characterizing the relationship between FEV_1_ (per 200 ml decrease) and cIMT across covariates of interest (n = 265)

| **Characteristic** | **Strata** | **Adjusted***  **β (95% CI)** | ***p* value** | ***p* value for FEV1*[covariate] interaction term**** |
| --- | --- | --- | --- | --- |
| Age | Age <55 (n = 173) | 0.004 (-0.001, 0.010) | 0.085 | 0.083 |
|  | Age ≥ 55 (n = 92) | 0.019 (0.008, 0.030) | 0.001 |  |
| Sex | Male (n = 140) | 0.007 (0.001, 0.012) | 0.019 | 0.812 |
|  | Female (n = 125) | 0.005 (-0.004, 0.014) | 0.240 |  |
| Smoking history | Never smokers (n = 134) | 0.006 (-0.001, 0.013) | 0.108 | 0.327 |
|  | Ever smokers (n = 131) | 0.007 (0.001, 0.013) | 0.018 |  |
| Socioeconomic status | Poorest (n = 65) | 0.008 (-0.001, 0.017) | 0.088 | 0.028 |
|  | Poor (n = 71) | 0.001 (-0.008, 0.010) | 0.890 |  |
|  | Less poor (n = 64) | 0.007 (-0.002, 0.017) | 0.140 |  |
|  | Least poor (n = 65) | 0.010 (0.000, 0.020) | 0.049 |  |
| HIV serostatus | HIV negative (n = 125) | 0.009 (0.001, 0.016) | 0.019 | 0.117 |
|  | HIV positive (n = 140) | 0.005 (-0.001, 0.011) | 0.099 |  |
| **Table S3 Legend**  Abbreviations: FEV_1_, forced expiratory volume in one second; cIMT, carotid intima media thickness; HIV, human immunodeficiency virus  Age and FEV_1_ were scaled as reported in the table and centered on median value of the cohort  *Models were stratified by the characteristic in each row and adjusted for the remaining variables  ***p* values derived from adjusted multivariable regression models in which a [covariate]*FEV_1_ product term was included instead of stratifying by the covariate of interest (e.g. p value for sex*FEV_1_ product term rather than stratifying by sex). | | | | |

**Tables S4 - S8:** Secondary analyses evaluating for heterogeneity in the relationship between cardiovascular disease and lung disease in models stratified by age (Table S4), sex (Table S5), smoking history (Table S6), socioeconomic status (Table S7), and HIV serostatus (Table S8).

|  | **Unadjusted** | | **Adjusted** | |
| --- | --- | --- | --- | --- |
| **Characteristic** | **β (95% CI)** | ***p* value** | **β (95% CI)** | ***p* value** |
|  | **Age < 55 (n = 173)** | | | |
| Female sex | 0.030 (0.004, 0.055) | 0.021 | 0.018 (-0.015, 0.051) | 0.279 |
| Ever smokers | -0.005 (-0.031, 0.020) | 0.679 | 0.006 (-0.020, 0.033) | 0.640 |
| Asset Index |  |  |  |  |
| Poorest | reference |  |  |  |
| Poor | 0.029 (0.002, 0.057) | 0.037 | 0.057 (0.022, 0.092) | 0.002 |
| Less poor | -0.016 (-0.046, 0.014) | 0.284 | 0.033 (-0.005, 0.070) | 0.088 |
| Least poor | 0.014 (-0.015, 0.044) | 0.333 | 0.052 (0.014, 0.090) | 0.002 |
| HIV positive | -0.016 (-0.042, 0.009) | 0.211 | -0.024 (-0.049, 0.002) | 0.070 |
| FEV_1_ (per 200 mL decrease) | 0.006 (0.002, 0.010) | 0.003 | 0.004 (-0.001, 0.010) | 0.085 |
|  | **Age ≥ 55 (n = 92)** | | | |
| Female sex | 0.033 (-0.010, 0.077) | 0.129 | -0.033 (-0.086, 0.021) | 0.226 |
| Ever smokers | -0.029 (-0.074, 0.015) | 0.193 | 0.001 (-0.046, 0.048) | 0.952 |
| Asset Index |  |  |  |  |
| Poorest | reference |  |  |  |
| Poor | 0.000 (-0.053, 0.053) | 0.996 | 0.009 (-0.051, 0.070) | 0.763 |
| Less poor | 0.026 (-0.025, 0.076) | 0.315 | 0.046 (-0.010, 0.102) | 0.105 |
| Least poor | 0.044 (-0.007, 0.094) | 0.090 | 0.064 (0.004, 0.124) | 0.038 |
| HIV positive | -0.016 (-0.059, 0.028) | 0.478 | -0.026 (-0.068, 0.016) | 0.228 |
| FEV_1_ (per 200 mL decrease) | 0.016 (0.008, 0.024) | <0.001 | 0.019 (0.008, 0.030) | 0.001 |
| **Table S4 Legend**  Abbreviations: cIMT, carotid intima media thickness; UGANDAC, Uganda non-communicable diseases and aging cohort; HIV, Human immunodeficiency virus; FEV1, forced expiratory volume in one second; mL, milliliters  Reference categories for categorical variables: Sex - male; Ever smokers – lifelong never smokers; Asset index –poorest quartile; HIV Serostatus - HIV negative.  FEV1 was scaled as reported in the table and centered on median value of the cohort | | | | |

|  | **Unadjusted** | | **Adjusted** | |
| --- | --- | --- | --- | --- |
| **Characteristic** | **β (95% CI)** | ***p* value** | **β (95% CI)** | ***p* value** |
|  | **Male sex (n = 140)** | | | |
| Age (per 5-year increase) | 0.030 (0.020, 0.040) | <0.001 | 0.028 (0.018, 0.038) | <0.001 |
| Ever smokers | -0.009 (-0.042, 0.024) | 0.591 | -0.006 (-0.036, 0.024) | 0.701 |
| Asset Index |  |  |  |  |
| Poorest | reference |  |  |  |
| Poor | 0.014 (-0.021, 0.050) | 0.428 | 0.042 (0.003, 0.082) | 0.036 |
| Less poor | 0.000 (-0.035, 0.035) | 0.988 | 0.040 (0.001, 0.080) | 0.047 |
| Least poor | 0.026 (-0.013, 0.064) | 0.189 | 0.060 (0.016, 0.104) | 0.008 |
| HIV positive | -0.000 (-0.032, 0.032) | 0.991 | -0.010 (-0.039, 0.019) | 0.507 |
| FEV_1_ (per 200 mL decrease) | 0.011 (0.005, 0.016) | <0.001 | 0.007 (0.001, 0.012) | 0.019 |
|  | **Female sex (n = 125)** | | | |
| Age (per 5-year increase) | 0.035 (0.022, 0.048) | <0.001 | 0.029 (0.016, 0.043) | <0.001 |
| Ever smokers | 0.025 (-0.012, 0.061) | 0.181 | 0.017 (-0.018, 0.051) | 0.339 |
| Asset Index |  |  |  |  |
| Poorest | reference |  |  |  |
| Poor | 0.016 (-0.023, 0.055) | 0.426 | 0.033 (-0.010, 0.076) | 0.131 |
| Less poor | 0.008 (-0.036, 0.052) | 0.706 | 0.024 (-0.023, 0.071) | 0.315 |
| Least poor | 0.017 (-0.022, 0.055) | 0.388 | 0.042 (-0.002, 0.087) | 0.063 |
| HIV positive | -0.036 (-0.070, -0.002) | 0.039 | -0.035 (-0.067, -0.004) | 0.030 |
| FEV_1_ (per 200 mL decrease) | 0.010 (0.001, 0.020) | 0.038 | 0.005 (-0.004, 0.014) | 0.240 |
| **Table S5 Legend**  Abbreviations: cIMT, carotid intima media thickness; UGANDAC, Uganda non-communicable diseases and aging cohort; HIV, Human immunodeficiency virus; FEV1, forced expiratory volume in one second; mL, milliliters  Reference categories for categorical variables: Ever smokers – lifelong never smokers; Asset index –poorest quartile; HIV Serostatus - HIV negative.  Age and FEV1 were scaled as reported in the table and centered on median value of the cohort | | | | |

|  | **Unadjusted** | | **Adjusted** | |
| --- | --- | --- | --- | --- |
| **Characteristic** | **β (95% CI)** | ***p* value** | **β (95% CI)** | ***p* value** |
|  | **Never smokers (n = 134)** | | | |
| Age (per 5-year increase) | 0.030 (0.017, 0.043) | <0.001 | 0.027 (0.014, 0.040) | <0.001 |
| Female sex | 0.017 (-0.017, 0.050) | 0.336 | -0.004 (-0.045, 0.037) | 0.843 |
| Asset Index |  |  |  |  |
| Poorest | reference |  |  |  |
| Poor | 0.040 (0.002, 0.078) | 0.039 | 0.055 (0.008, 0.103) | 0.022 |
| Less poor | -0.040 (-0.078, -0.002) | 0.037 | 0.004 (-0.045, 0.052) | 0.879 |
| Least poor | 0.020 (-0.015, 0.055) | 0.258 | 0.038 (-0.007, 0.084) | 0.094 |
| HIV positive | -0.023 (-0.056, 0.010) | 0.174 | -0.017 (-0.048, 0.015) | 0.290 |
| FEV_1_ (per 200 mL decrease) | 0.008 (0.002, 0.013) | 0.007 | 0.006 (-0.001, 0.013) | 0.108 |
|  | **Ever Smokers (n = 131)** | | | |
| Age (per 5-year increase) | 0.033 (0.022, 0.043) | <0.001 | 0.029 (0.020, 0.039) | <0.001 |
| Female sex | 0.050 (0.015, 0.086) | 0.006 | 0.026 (-0.010, 0.062) | 0.154 |
| Asset Index |  |  |  |  |
| Poorest | reference |  |  |  |
| Poor | -0.007 (-0.044, 0.030) | 0.696 | 0.018 (-0.017, 0.053) | 0.312 |
| Less poor | 0.041 (0.002, 0.081) | 0.040 | 0.059 (0.022, 0.095) | 0.002 |
| Least poor | 0.032 (-0.016, 0.080) | 0.189 | 0.066 (0.022, 0.110) | 0.003 |
| HIV positive | -0.013 (-0.047, 0.021) | 0.436 | -0.023 (-0.051, 0.005) | 0.112 |
| FEV_1_ (per 200 mL decrease) | 0.012 (0.007, 0.018) | <0.001 | 0.007 (0.001, 0.013) | 0.018 |
| **Table S6 Legend**  Abbreviations: cIMT, carotid intima media thickness; UGANDAC, Uganda non-communicable diseases and aging cohort; HIV, Human immunodeficiency virus; FEV1, forced expiratory volume in one second; mL, milliliters  Reference categories for categorical variables: Sex - male; Asset index –poorest quartile; HIV Serostatus - HIV negative.  Age and FEV1 were scaled as reported in the table and centered on median value of the cohort | | | | |

|  | **Unadjusted** | | **Adjusted** | |
| --- | --- | --- | --- | --- |
| **Characteristic** | **β (95% CI)** | ***p* value** | **β (95% CI)** | ***p* value** |
|  | **Lowest (Q1) Asset Index Quartile - poorest (n = 65)** | | | |
| Age (per 5-year increase) | 0.027 (0.012, 0.042) | 0.001 | 0.029 (0.014, 0.044) | <0.001 |
| Female | 0.033 (-0.008, 0.073) | 0.115 | 0.026 (-0.025, 0.076) | 0.314 |
| Ever Smokers | -0.009 (-0.052, 0.035) | 0.692 | 0.006 (-0.035, 0.048) | 0.762 |
| HIV positive | 0.012 (-0.030, 0.053) | 0.582 | -0.007 (-0.045, 0.030) | 0.698 |
| FEV_1_ (per 200 mL decrease) | 0.010 (0.003, 0.018) | 0.006 | 0.008 (-0.001, 0.017) | 0.088 |
|  | **Q2 Asset Index - poor (n = 71)** | | | |
| Age (per 5-year increase) | 0.023 (0.008, 0.037) | 0.002 | 0.020 (0.005, 0.034) | 0.008 |
| Female | 0.032 (-0.014, 0.077) | 0.167 | 0.028 (-0.024, 0.079) | 0.287 |
| Ever Smokers | -0.039 (-0.084, 0.006) | 0.091 | -0.039 (-0.082, 0.004) | 0.076 |
| HIV positive | -0.061 (-0.105, -0.017) | 0.007 | -0.053 (-0.096, -0.010) | 0.017 |
| FEV_1_ (per 200 mL decrease) | 0.005 (-0.003, 0.013) | 0.247 | 0.001 (-0.008, 0.010) | 0.890 |
|  | **Q3 Asset Index - less poor (n = 64)** | | | |
| Age (per 5-year increase) | 0.045 (0.027, 0.062) | <0.001 | 0.037 (0.019, 0.055) | <0.001 |
| Female | 0.037 (-0.015, 0.090) | 0.163 | 0.019 (-0.047, 0.085) | 0.560 |
| Ever Smokers | 0.059 (0.009, 0.108) | 0.021 | 0.052 (0.007, 0.096) | 0.024 |
| HIV positive | 0.002 (-0.050, 0.054) | 0.936 | 0.015 (-0.030, 0.059) | 0.513 |
| FEV_1_ (per 200 mL decrease) | 0.011 (0.004, 0.018) | 0.003 | 0.007 (-0.002, 0.017) | 0.140 |
|  | **Highest (Q4) Asset Index Quartile - least poor (n = 65)** | | | |
| Age (per 5-year increase) | 0.035 (0.016, 0.053) | <0.001 | 0.028 (0.009, 0.047) | 0.005 |
| Female | 0.023 (-0.027, 0.072) | 0.370 | -0.012 (-0.068, 0.044) | 0.664 |
| Ever Smokers | 0.011 (-0.044, 0.066) | 0.686 | 0.016 (-0.036, 0.068) | 0.539 |
| HIV positive | -0.049 (-0.104, 0.005) | 0.075 | -0.037 (-0.087, 0.014) | 0.151 |
| FEV_1_ (per 200 mL decrease) | 0.012 (0.003, 0.021) | 0.009 | 0.010 (0.000, 0.020) | 0.049 |
| **Table S7 Legend**  Abbreviations: HIV, Human immunodeficiency virus; FEV1, forced expiratory volume in one second; mL, milliliters  Reference categories for categorical variables: Sex - male; Ever smokers – lifelong never smokers; HIV Serostatus - HIV negative.  Age and FEV_1_ were scaled as reported in the table and centered on median value of the cohort | | | | |

|  | **Unadjusted** | | **Adjusted** | |
| --- | --- | --- | --- | --- |
| **Characteristic** | **β (95% CI)** | ***p* value** | **β (95% CI)** | ***p* value** |
|  | **HIV-negative (n = 125)** | | | |
| Age (per 5-year increase) | 0.031 (0.021, 0.042) | <0.001 | 0.024 (0.014, 0.035) | <0.001 |
| Female sex | 0.049 (0.017, 0.081) | 0.003 | 0.022 (-0.018, 0.063) | 0.278 |
| Ever Smokers | -0.010 (-0.044, 0.023) | 0.540 | 0.014 (-0.015, 0.043) | 0.343 |
| Asset Index |  |  |  |  |
| Poorest | reference |  |  |  |
| Poor | -0.062 (-0.097, -0.027) | 0.001 | -0.069 (-0.113, -0.025) | 0.002 |
| Less poor | 0.041 (0.006, 0.076) | 0.022 | -0.011 (-0.054, 0.031) | 0.602 |
| Least poor | -0.014 (-0.053, 0.026) | 0.486 | -0.041 (-0.087, 0.004) | 0.076 |
| FEV1 (per 200 mL decrease) | 0.014 (0.009, 0.020) | <0.001 | 0.009 (0.001, 0.016) | 0.019 |
|  | **HIV-positive (n = 140)** | | | |
| Age (per 5-year increase) | 0.030 (0.018, 0.043) | 0.000 | 0.029 (0.016, 0.042) | <0.001 |
| Female sex | 0.013 (-0.020, 0.047) | 0.427 | 0.004 (-0.035, 0.042) | 0.849 |
| Ever Smokers | -0.001 (-0.034, 0.033) | 0.961 | 0.001 (-0.033, 0.035) | 0.937 |
| Asset Index |  |  |  |  |
| Poorest | reference |  |  |  |
| Poor | -0.018 (-0.060, 0.023) | 0.390 | -0.038 (-0.082, 0.007) | 0.099 |
| Less poor | -0.021 (-0.061, 0.019) | 0.307 | -0.023 (-0.067, 0.020) | 0.293 |
| Least poor | 0.012 (-0.026, 0.050) | 0.540 | -0.005 (-0.046, 0.037) | 0.819 |
| FEV1 (per 200 mL decrease) | 0.007 (0.001, 0.012) | 0.017 | 0.005 (-0.001, 0.011) | 0.099 |
| **Table S8 Legend**  Abbreviations: HIV, Human immunodeficiency virus; FEV1, forced expiratory volume in one second; mL, milliliters  Reference categories for categorical variables: Sex - male; Ever smokers – lifelong never smokers; Asset index –poorest quartile.  Age and FEV_1_ were scaled as reported in the table and centered on median value of the cohort | | | | |

**Table S9:** Sensitivity analysis evaluating correlates of increased cIMT in the UGANDAC cohort using FEV1/FVC as the lung function metric of choice rather than FEV1 (n = 265).

|  | **Unadjusted** | | **Adjusted** | |
| --- | --- | --- | --- | --- |
| **Characteristic** | **β (95% CI)** | ***p* value** | **β (95% CI)** | ***p* value** |
| Age (per 5-year increase) | 0.031 (0.023, 0.039) | <0.001 | 0.030 (0.022, 0.038) | <0.001 |
| Female | 0.031 (0.007, 0.054) | 0.010 | 0.036 (0.014, 0.057) | 0.001 |
| Ever Smokers | -0.003 (-0.027, 0.020) | 0.794 | 0.002 (-0.020, 0.025) | 0.849 |
| Asset Index |  |  |  |  |
| Poorest | reference |  |  |  |
| Poor | -0.039 (-0.066, -0.012) | 0.005 | -0.052 (-0.083, -0.021) | 0.001 |
| Less poor | 0.015 (-0.012, 0.041) | 0.275 | -0.013 (-0.043, 0.017) | 0.392 |
| Least poor | -0.000 (-0.028, 0.027) | 0.978 | -0.018 (-0.048, 0.012) | 0.229 |
| HIV positive | -0.018 (-0.041, 0.006) | 0.142 | -0.022 (-0.043, -0.000) | 0.047 |
| FEV_1_/FVC (per 10% decrease in ratio) | 0.019 (0.000, 0.038) | 0.044 | 0.013 (-0.004, 0.029) | 0.145 |
| **Table S9 Legend**  Abbreviations: cIMT, carotid intima media thickness; HIV, Human immunodeficiency virus; FEV_1_, forced expiratory volume in one second; mL, milliliters  Reference categories for categorical variables: Sex - male; Ever smokers – lifelong never smokers; Asset index –poorest quartile; HIV Serostatus - HIV negative.  Age and FEV_1_/FVC were scaled as reported in the table and centered on median value of the cohort | | | | |

**Table S10:** Sensitivity analysis evaluating correlates of increased cIMT in the UGANDAC cohort including physical activity in the model (n = 261).

|  | **Unadjusted** | | **Adjusted** | |
| --- | --- | --- | --- | --- |
| **Characteristic** | **β (95% CI)** | ***p* value** | **β (95% CI)** | ***p* value** |
| Age (per 5-year increase) | 0.031 (0.023, 0.039) | <0.001 | 0.029 (0.020, 0.037) | <0.001 |
| Female | 0.031 (0.007, 0.054) | 0.010 | 0.010 (-0.018, 0.038) | 0.482 |
| Activity per week (per 120 min increase) | -0.003 (-0.027, 0.020) | 0.794 | 0.003 (-0.020, 0.025) | 0.815 |
| Ever Smokers | 0.000 (-0.000, 0.001) | 0.205 | 0.000 (-0.000, 0.001) | 0.346 |
| Asset Index |  |  |  |  |
| Poorest |  |  |  |  |
| Poor | -0.039 (-0.066, -0.012) | 0.005 | -0.052 (-0.084, -0.020) | 0.001 |
| Less poor | 0.015 (-0.012, 0.041) | 0.275 | -0.018 (-0.048, 0.013) | 0.253 |
| Least poor | -0.000 (-0.028, 0.027) | 0.978 | -0.018 (-0.049, 0.012) | 0.239 |
| HIV positive | -0.018 (-0.041, 0.006) | 0.142 | -0.019 (-0.041, 0.002) | 0.079 |
| FEV1 (per 200 mL decrease) | 0.010 (0.006, 0.014) | <0.001 | 0.006 (0.001, 0.011) | 0.011 |
| **Table S10 Legend**  Abbreviations: cIMT, carotid intima media thickness; HIV, Human immunodeficiency virus; FEV_1_, forced expiratory volume in one second; mL, milliliters  Reference categories for categorical variables: Sex - male; Ever smokers – lifelong never smokers; Asset index –poorest quartile; HIV Serostatus - HIV negative.  Age, Activity, and FEV_1_ were scaled as reported in the table and centered on median value of the cohort | | | | |

**Figure S1:** Forest plot of the relationships between lung function (per 200mL decrease in FEV_1_) and cIMT (in millimeters), stratified by covariates of interest


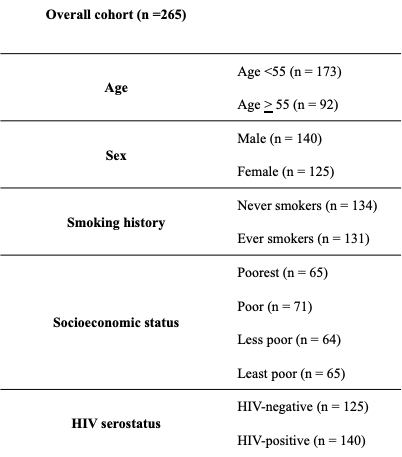


**Figure S1 Legend:**

Abbreviations: *n,* number; β, beta coefficient; HIV, human immunodeficiency virus

**Figure S2.** **The relationship between FEV1 and cIMT among PLWH as compared to HIV uninfected individuals.** FEV_1_: forced expiratory volume in one second; cIMT: carotid intima media thickness
